# Utilizing in ovo telemetry to examine the effects of reduced incubation temperature on broiler embryo temperature and subsequent hatchability

**DOI:** 10.1016/j.psj.2023.102506

**Published:** 2023-01-16

**Authors:** L.L. Lindsey, K.E.C. Elliott, S.A. Fatemi, P.D. Gerard, E.D. Peebles

**Affiliations:** ⁎ Department of Poultry Science, Mississippi State University, Mississippi State MS 39762, USA; † USDA-ARS, Poultry Research Unit, Mississippi State, MS 39762, USA; ‡ School of Mathematical and Statistical Sciences, Clemson University, Clemson, SC 29634, USA

**Keywords:** broiler, embryo, hatchability, incubation, transponder

## Abstract

The current commercial broiler embryo experiences a rapid metabolism and growth rate and may produce more heat than those of the past. Consequently, it may be beneficial to lower standard incubation temperature for commercial broiler hatching eggs. The purpose of this experiment was to determine if lowering incubation temperature at 12 d of incubation (**DOI**) would affect embryo temperature (**ET**) in embryonated Ross 708 broiler hatching eggs. From 0 to 12 DOI, eggs were incubated under standard conditions (37.50°C dry bulb, 29.76°C wet bulb). At 12 DOI, temperature transponders were aseptically placed on the inner air cell membrane to measure air cell temperature (**ACT**) as an estimate of ET in 120 eggs. The eggs were then randomly assigned to 4 separate still-air incubators, each containing 30 eggs. Two replicate incubators were maintained at a standard (**STRT**; 37.5°C) or low (**LTRT**; 35.6°C) temperature treatment between 12 and 21 DOI. A significant positive correlation existed between incubator air temperature (**IAT**) and ACT across temperature treatment and in the STRT. However, IAT was not significantly correlated with ACT in the LTRT. A significantly higher ACT was observed in the STRT than in the LTRT for 88% of the readings taken between 12 and 21 DOI. Percent egg weight loss (**PEWL**) between 13 and 17 DOI did not significantly differ between temperature treatments. From 13 to 17 DOI, there was a significant positive correlation between PEWL and ACT in the STRT, however, no significant correlation occurred between PEWL and ACT in the LTRT. Percent hatch of fertile eggs containing live embryos at 12 DOI was 93.3% in the STRT and 100% in the LTRT. However, time of hatch occurred 14 to 19 h later and hatchling BW was lower in the LTRT than in the STRT. Although lowering IAT at 12 DOI may improve hatchability, an associated delay in hatch and a decrease in hatchling BW may not be commercially acceptable.

## INTRODUCTION

Modern broilers are genetically selected to grow faster and produce more heat than broilers of the past (Havenstein et al., 1994; Hulet, 2007; Collins et al., 2014). Hamidu et al. (2018) found that genetic selection over time has led to an increase in commercial broiler embryo metabolism. Because the current embryo in commercial broiler hatching eggs has a rapid rate of growth and metabolism, it subsequently has a higher level of metabolic heat production. Of the various factors that affect hatchability, one of the most critical is incubation temperature (Wilson, 1991; Hulet, 2007), with deviations from an optimal temperature having an impact on embryo development and overall hatching success (Wilson, 1991). Even slight deviations (less than 1.0°C) can affect hatchability and chick quality (Lourens et al., 2005). Therefore, incubator air temperature (**IAT**) may need to be adjusted to accommodate higher levels of embryo heat production. Since embryonic growth represents 30 to 40% of the total development time of the commercial broiler (Hulet, 2007), incubation conditions can also influence posthatch broiler performance (Clark et al., 2017).

Incubation conditions that are recommended for today's commercial broiler hatching eggs include an IAT of 37.5 to 37.8°C (Bruzual et al., 2000; Almeida et al., 2016; Hamidu et al., 2018) and a relative humidity of 53 to 55% (Bruzual et al., 2000). Nevertheless, IAT is not the same as the temperature experienced by the embryo (**ET**) (French, 1997; Lourens et al., 2005). Joseph et al. (2006) stated that ET has a greater influence on hatchability and embryonic development than IAT. French (2009a) has aptly stated that what the embryo experiences inside the egg should be considered the actual incubation temperature, because that is the temperature that determines the development of the embryo. Consequently, hatchability and the development of the embryo are more influenced by ET rather than by IAT (Lourens et al., 2005). Heat production from the metabolism of the embryo is a major determining factor of ET (French, 2009b). As embryos age, heat production increases inside the egg, which subsequently increases ET (Barott, 1937; French, 1997). During the later half of incubation, metabolic heat production by the embryo also leads to an increase in IAT (French, 1997). Because ET has been defined as a combination of IAT, the thermal conductance of the eggshell, and the heat that the embryo produces (French, 1997), these factors together create a complex and dynamic system that determine the temperature that the embryo experiences (French, 2009b). Embryo temperature has been estimated by measuring eggshell temperature (**EST**) (Lourens et al., 2005). However, Olojede et al. (2017) found that air cell temperatures can be significantly higher than EST. Peebles et al. (2012) concluded that air cell temperature (**ACT**) readings detect not only a higher temperature, but also a temperature that is closer to that of actual ET than that of EST. An EST reading may, therefore, not be the most accurate and precise means by which to determine the temperature that is experienced by the embryo (Olojede et al., 2017; Hamidu et al., 2018).

Larger broiler breeder hens produce larger eggs (Fletcher et al., 1983), and the hatchability of fertile eggs can decrease with an increase in egg size (Elibol and Brake, 2008), because eggs of different mass can be affected differently by IAT (French, 1997; Elibol and Brake, 2008). A higher level of heat production by commercial broiler embryos further exacerbates other factors, such as egg size and egg location within the incubator that can influence egg temperature. With these factors present, broiler chicks may be experiencing higher temperatures inside the egg than expected. This can lead to an overheating of embryos, causing them to be compromised in quality (Hulet, 2007). Thus, lowering IAT at various periods during incubation could prove to be beneficial to both broiler embryos and hatched chicks. Hypothermic and hyperthermic effects depend on embryonic age, and the length and extent of exposure to environmental temperature change (Wilson, 1991). Embryos tend to be more susceptible to nonoptimal temperatures during the early stages of incubation (Romanoff, 1936), and the Ross 708 broiler, selected for higher breast meat yields, tends to be more sensitive to changes in incubation temperature than other strains, such as the Ross 308 broiler (Hamidu et al., 2018).

Ipek et al. (2015) reported that when the EST of Cobb 500 broiler hatching eggs was lowered from an average of 38.0°C to an average of 35.0°C from 0 to 18 d of incubation (**DOI**) that posthatch BW through 6 wk of age was significantly decreased. Nevertheless, implementation of a more restricted decrease in the IAT of hatching eggs from a faster growing broiler strain during a later DOI period may yield some positive results in performance. Almeida et al. (2016) found that hatchability of fertile eggs and hatchling yolk-free BW was not significantly different when Cobb 500 broiler hatching eggs were incubated at 36.0°C or 37.5°C. Therefore, 35.6°C was considered as a viable temperature for the LTRT treatment. Pulikanti et al. (2011) found that the earliest time a temperature-recording transponder could be placed in the air cell without negatively affecting embryo survivability was at 12 DOI. That method has been tested and is considered safe without causing adverse effects to the embryo (Peebles et al., 2012; Olojede et al., 2017). Lying on the inner eggshell membrane overlaying the embryo, a transponder's proximity to the embryo establishes ACT readings as being potentially more accurate than EST in estimating actual ET (Peebles et al., 2012; Olojede et al., 2017). Thus, the use of implantable temperature transponders has the potential of being a good alternate method by which to estimate ET more accurately and precisely. With this technology in mind, the purpose of this experiment was to determine if a lower IAT, initiated at 12 DOI, would have a direct effect on embryonated Ross 708 broiler hatching egg ACT. This was accomplished using temperature transponders and a wireless probe, with the transponder resting on the inner eggshell membrane of the air cell.

## MATERIALS AND METHODS

All handling and care of broiler hatchlings was conducted under the approval of the Mississippi State University Institutional Animal Care and Use Committee (Protocol # IACUC-20-248). Ross 708 broiler hatching eggs were obtained from 41-wk-old hens from a commercial source, and they were stored under commercial conditions according to the procedure of Fatemi et al. (2020). Eggs that were mis-shaped, contaminated, or that had cracked, or malformed shells were discarded and not set for incubation (Pulikanti et al., 2011). Between 0 and 12 DOI, approximately 540 of the Ross 708 broiler hatching eggs were incubated under standard conditions (37.5°C and 55% relative humidity) in a Nature Form incubator (NOM 1080, Jacksonville, FL). The air temperature and relative humidity within the incubator were also monitored and maintained throughout the incubational period in accordance with the method described by Fatemi et al. (2020). At 12 DOI, 120 eggs were randomly selected and candled for verification of live embryonation. Following the procedures of Pulikanti et al. (2011), a transponder (implantable, programmable, temperature transponder; IPTT- 300; Bio Medic Data Systems Inc., Seaford, DE. Accuracy ± 0.2°C) measuring 14 mm in length and 2 mm in diameter, was inserted aseptically into each air cell, allowing it to rest on the inner eggshell membrane next to the body of the embryo. As specified by Pulikanti et al. (2011), holes were subsequently sealed with an adhesive that allowed for gas exchange.

At 12 DOI, the 120 live embryonated eggs were divided into groups of 4 (30 eggs each) and placed in 4 separate 1602 N-Thermal Air Hovabator incubators (GQF Manufacturing Co., Inc., Savannah, GA) that occupied the same room. Two replicate incubators were each assigned a treatment of 37.5°C (standard treatment, **STRT**) or 35.6°C (low treatment, **LTRT**), which were maintained at those settings through 21 DOI. Mean relative humidity in the incubators belonging to the STRT and LTRT treatments were 58.00% and 59.08%, respectively. The Hovabator incubators were chosen due to their Styrofoam walls, which allowed for transponders within the air cell of each egg oriented near the Styrofoam wall, to be read wirelessly without opening the incubators. HOBO ZW data loggers (HOBO ZW series wireless, Onset Computer Corporation, Bourne, MA. Accuracy ± 0.21°C) were used to record room temperature, IAT, and incubator relative humidity. Logger readings were recorded each min, and the IAT of the incubators was adjusted according to treatment designation, as necessary. One HOBO ZW data logger stopped recording on 19 DOI at 10:00 PM. Therefore, IAT readings only at times from 12 DOI at 12:00 PM to 19 DOI at 10:00 PM were recorded. The mean IAT for Day 12 was based on readings taken every min between 12:00 PM and 11:59 PM. The mean IAT for Days 13 to 18 were based on readings taken every min between 12:00 AM and 11:59 PM. Room temperature and IAT readings in each incubator continued until all chicks in an incubator had hatched.

Recordings of ACT were taken from 12 DOI at 12:00 PM to 21 DOI at 10:00 PM. There were 3 daily recordings from 12 to 18 DOI, 4 daily recordings at 19 DOI, and 5 daily recordings in the 20 to 21 DOI period (Figure 4, Figure 5, Figure 6). The ACT recordings were made using telemetric technology, involving a wireless probe and associated software (DAS-6006/7 Smart Probe, Bio Medic Data Systems Inc., Seaford, DE). The wireless probe was used to scan the transponder of each egg, with each egg laying near the wall of the incubator for the accurate recording of ACT. The ACT readings were taken thrice daily, 5 h apart, (7:00 AM, 12:00 PM, and 5:00 PM) from 12 to 18 DOI. The total duration of the ACT readings for all 120 eggs was approximately 30 min. Following the ACT transponder readings, eggs were turned by hand thrice daily through 18 DOI, maintaining air cell orientation near the Styrofoam wall, and were weighed individually at 13, 15, and 17 DOI. Egg turning caused only minor transitory changes in IAT. Eggs were weighed on 13, 15, and 17 DOI. Mean egg weight at 13 DOI within the STRT and LTRT treatments are provided in Table 1. Percentage egg weight loss **(PEWL)** was calculated for the 13 to 15, 15 to 17, and 13 to 17 DOI periods. The PEWL of each egg was calculated according to the procedure of Peebles et al. (2001). Additional ACT readings were taken at 10:00 PM and 3:00 AM daily after 18 DOI until the hatching process for each individual egg was completed.

**Table 1:** Mean egg weight at 13 days of incubation (DOI), and hatchling BW within the 37.5°C (STRT) and 35.6°C (LTRT) treatments.

Treatment	13 DOI egg weight (g)	Hatchling BW (g)
STRT	61.2	50.0^a^
LTRT^1^	61.3	47.1^b^
SEM	0.78	0.76
P-value	0.831	0.001

a,b Treatment means within the same variable column with no common superscripts are significantly different (*P* < 0.05).

1 Eggs in the LTRT treatment were incubated at 37.5°C from 0 to 12 DOI, and then at 35.6°C from 12 to 18 DOI.

At 18 DOI, manual egg turning ceased, and each egg was enveloped in sheer mesh fabric to retain each transponder at hatch. In each replicate incubator in each treatment, the non-cumulative and cumulative mean hatch time (**HT**) profiles [between 20.3 (487 h) and 22.3 (535 h) DOI] of viable chicks were recorded. A chick was considered as being viable when its down was dry and was mobile and fully hatched at 22.3 DOI. Hatchability (**HVC**) at 22.3 DOI was calculated as the number of viable chicks as a percentage of the number of eggs determined to contain live embryos at 12 DOI. At 18 DOI and after, the incubators were opened only after ACT readings were taken on the remaining unhatched eggs, and chicks remained in the incubators until all chicks were pulled from their respective incubator by 22.3 DOI. The BW of each hatchling at pull was determined, and mean hatchling BW within the STRT and LTRT treatments are provided in Table 1. All unhatched eggs were opened for egg residue analysis using the procedures of Hamburger and Hamilton (1951) as a reference for descriptions of embryo characteristics at the early, middle, and late stages of incubation. Any chick that hatched with an unhealed navel or with externalized intestines was recorded as a cull and was humanely euthanized.

### Statistical Analysis

There were 2 replicate incubators per treatment, with incubator and individual egg or bird considered as random effects. The effects of temperature treatment on ACT, and differences between IAT and ACT across and within each temperature treatment group, were analyzed using repeated measures analysis. The above analyses, including the effect of temperature treatment on PEWL between 13 to 15, 15 to 17, and 13 to 17 DOI, and the effects of temperature treatment on HT were tested using Proc MIXED. Differences between STRT and LTRT for HVC were not statistically compared, because of having respective values of 93.3% and 100%. Least squares means were compared using Fisher's Protected LSD test in the event of significant global effects. Linear regression analysis was performed for daily means of ACT in the STRT and the LTRT using Proc Reg to compute line parameter and R^2^ values. Partial correlation analyses between IAT and ACT across and within temperature treatment, and between PEWL and ACT within the 13 to 15, 15 to 17, and 13 to 17 DOI time periods were performed using MANOVA of Proc GLM. All statistical analyses were carried out using SAS version 9.4 (SAS Institute, 2013; Fatemi et al., 2022), and all tests were considered significant at P ≤ 0.05.

## RESULTS

### Incubator Air Temperature

**Figure 1:**
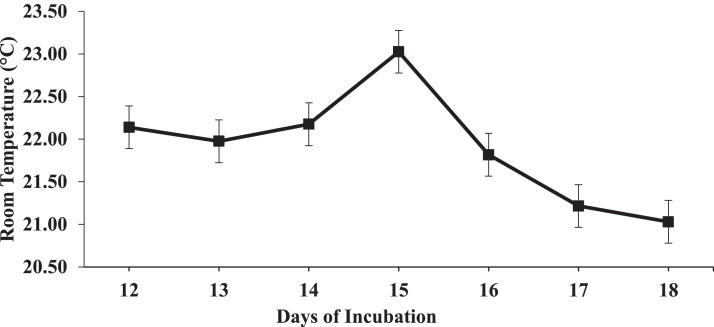
Mean daily room air temperature from 12 to 18 d of incubation (DOI). Daily means were calculated from readings taken every min from 12:00 PM to 11:59 PM on 12 DOI, and from 12:00 AM to 11:59 PM in the 13 to 18 DOI time periods.

**Figure 2:**
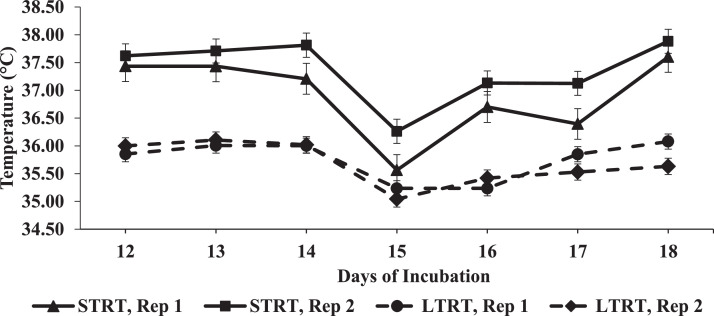
Mean daily incubator air temperatures for each individual replicate incubator in the 37.5°C (STRT) and 35.6°C (LTRT) treatments between 12 and 18 d of incubation (DOI). Eggs in the STRT and LTRT were incubated at 37.5°C from 0 to 12 DOI.

**Figure 3:**
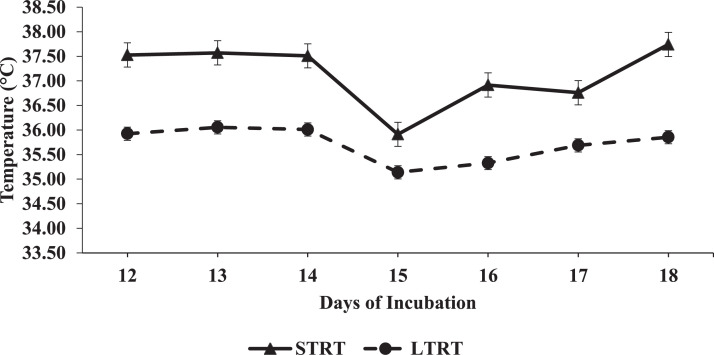
Mean daily incubator air temperatures for the 37.5°C (STRT) and 35.6°C (LTRT) treatments between 12 and 18 d of incubation (DOI). Eggs in the STRT and LTRT were incubated at 37.5°C from 0 to 12 DOI.

Between 12 and 18 DOI, daily room temperature means are presented in Figure 1, daily IAT means of each individual replicate incubator within each treatment are presented in Figure 2, and daily IAT means across the replicate incubators for each treatment are presented in Figure 3. In the 12 to 18 DOI period, the mean IAT of the replicate incubators that belonged to the 37.5°C (STRT) treatment were 36.9°C and 37.4°C, and of the replicate incubators that belonged to the 35.6°C (LTRT) treatment were 35.8°C and 35.7°C. The IAT readings for all 4 incubators were consistently close to their set temperatures. The mean IAT of the 2 replicate STRT incubators was lowest at 15 DOI (Figure 2), which may be related to a counter response of the incubator thermostat to the higher room air temperature on that day (Figure 1).

There was an approximate 0.46°C difference between the mean IAT for each of the 2 replicate incubators belonging to the STRT, with the greatest difference being 0.73°C (Figure 2), and there was an approximate 0.07°C difference between the mean IAT of the 2 replicate incubators belonging to the LTRT, with the greatest difference being 0.45°C (Figure 2). The replicate incubators belonging to the 37.5°C treatment had a correlation coefficient of 0.97242 and the replicate incubators belonging to the 35.6°C treatment had a correlation coefficient of 0.78284. The IAT of each of the 4 incubators fluctuated marginally. As shown in Figure 2, the IAT for the 2 replicate incubators belonging to the STRT remained different from the 2 replicate incubators of the LTRT regardless of their individual fluctuations. Time and temperature treatment interacted significantly (*P* < 0.0001) to affect IAT (Figure 3), signifying that the difference in IAT between the STRT and LTRT changed with time. Overall, the IAT for each treatment was maintained near the set temperature, with an approximate 1.42°C mean difference between the treatments (Figure 3).

### Air Cell Temperature

**Figure 4:**
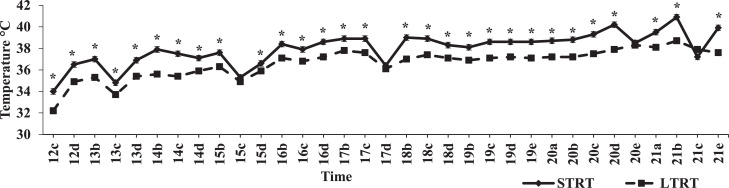
Mean air cell temperature in the 37.5°C (STRT) and 35.6°C (LTRT) treatments at recorded times from 12 to 21 d of incubation (DOI). “a” = 3:00 AM, “b” = 7:00 AM, “c” = 12:00 PM, “d” = 5:00 PM, and “e” = 10:00 PM. A ‘*’ indicates a significant (*P* ≤ 0.05) treatment difference. Pooled SEM for the STRT and LTRT were 0.165°C and 0.162°C, respectively. Eggs in the STRT and LTRT were incubated at 37.5°C from 0 to 12 DOI. Line parameters = STRT (y = 0.186x + 36.14) and LTRT (y = 0.219x + 34.64).

Time and temperature treatment interacted significantly (*P* < 0.0001) to affect ACT, and like IAT, would signify that the difference between the treatment means changed over time between 12 and 21 DOI (Figure 4). From 12 to 21 DOI, mean ACT for the STRT was 37.98°C (Pooled SEM = 0.165°C) and for the LTRT was 36.61°C (Pooled SEM = 0.162°C; Figure 4). Linear regression analyses of ACT in the STRT (R^2^ = 0.4740; *P* < 0.0001) and LTRT (R^2^ = 0.6597; *P* < 0.0001) were significant. These analyses indicate that ACT in the STRT and LTRT exhibited gradual linear increases over time, with positive slope values being 0.186 and 0.219 for the STRT and LTRT, respectively. In comparison to the LTRT, most ACT readings in the STRT were significantly (*P* ≤ 0.05) higher from 12 to 21 DOI, except for the 15 DOI at 12:00 PM, 17 DOI at 5:00 PM, 20 DOI at 10:00 PM, and 21 DOI at 12:00 PM time periods (Figure 4).

**Figure 5:**
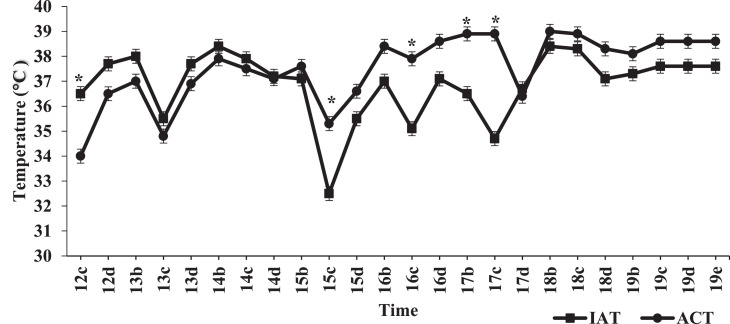
Mean daily incubator air (IAT) and air cell temperature (ACT) readings in the 37.5°C treatment (STRT) at recorded times between 12 and 19 d of incubation. “b” = 7:00 AM, “c” = 12:00 PM, “d” = 5:00 PM, and “e” = 10:00 PM. A ‘*’ indicates a significant (*P* ≤ 0.05) difference between ACT and IAT. The STRT eggs were incubated at 37.5°C from 0 to 12 DOI.

**Figure 6:**
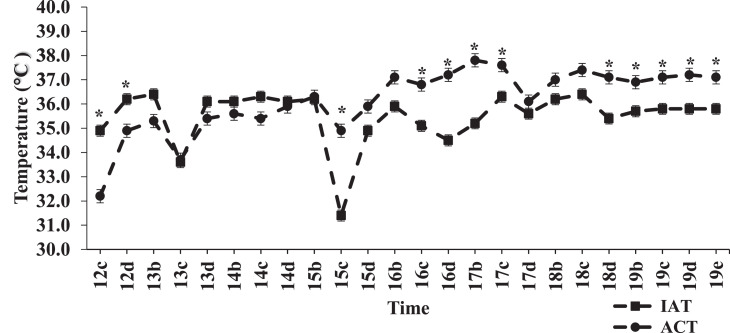
Mean daily incubator air (IAT) and air cell temperature (ACT) readings in the 35.6°C treatment (LTRT) at recorded times between 12 and 19 d of incubation (DOI). “b” = 7:00 AM, “c” = 12:00 PM, “d” = 5:00 PM, and “e” = 10:00 PM. A ‘*’ indicates a significant (*P* ≤ 0.05) difference between ACT and IAT. The LTRT eggs were incubated at 37.5°C from 0 to 12 DOI.

Comparisons between IAT and ACT at various time periods between 12 and 19 DOI in the STRT and LTRT are shown in Figures 5 and 6, respectively. For both treatments, initial ACT readings were lower than that of the IAT. The low ACT values in the STRT (Figure 5) and LTRT (Figure 6) observed at 12:00 PM on 12 DOI corresponded with egg transfer. However, at approximately 15 DOI, the ACT of both treatments numerically rose above IAT, and except for 5:00 PM on 17 DOI, remained above IAT through 19 DOI. Time and location of temperature reading (IAT and ACT) interacted significantly (*P* < 0.0001) across and within temperature treatment (STRT and LTRT). In the STRT, ACT was significantly higher than IAT a total of 4 out of 24 times (Figure 5), whereas in the LTRT, ACT was significantly higher than IAT a total of 10 out of 24 times (Figure 6). Across temperature treatment, significant differences between IAT and ACT followed the same pattern as those noted for the STRT (Figure 5), except that at 5:00 PM on 16 DOI, ACT was also significantly higher than IAT across temperature treatment. Within the LTRT, IAT and ACT were not significantly (*P* = 0.455) correlated (r = −0.0203). However, there was a significant (*P* = 0.001) positive correlation (r = 0.064) between IAT and ACT across treatment. Moreover, although IAT and ACT in the LTRT were not significantly correlated, there was a significant (*P* < 0.0001) positive correlation (r = 0.2311) between IAT and the ACT in the STRT, indicating that as IAT increased in the STRT, so did ACT.

### Percentage Egg Weight Loss

Egg weight at 13 DOI was not significantly different between the STRT and LTRT treatments (Table 1), and there was no significant effect of IAT on PEWL in the 13 to 15, 15 to 17, or 13 to 17 DOI intervals (*P* = 0.871, 0.314, and 0.834, for 13 to 15 DOI, 15 to 17 DOI, and 13 to 17 DOI, respectively). In the 13 to 17 DOI interval, mean PEWL in the STRT and LTRT were 2.4%, and 2.0%, respectively. The results of correlation analyses between PEWL and ACT across and within temperature treatment in the 13 to 15, 15 to 17, and 13 to 17 DOI intervals are shown in Table 2. Across treatment, there was a significant positive correlation between PEWL and ACT in the 15 to 17 DOI time interval. Also, within the STRT, there was a significant positive correlation between PEWL and ACT in the 13 to 15 DOI and 13 to 17 DOI time intervals, with marginal significance in the 15 to 17 DOI time interval. However, there was no significant correlation between PEWL and ACT in any of the 3 DOI intervals in the LTRT.

**Table 2:** Correlations between percentage egg weight loss and air cell temperature within 13 to 15, 15 to 17, and 13 to 17 days of incubation (DOI) time periods across and within the 37.5°C (STRT) and 35.6°C (LTRT) treatments^1^.

Time period	Across treatment	STRT	LTRT
13 to 15 DOI	r = 0.076874 (*P* = 0.4162)	r = 0.355377 (*P* = 0.0078)	r = 0.031295 (*P* = 0.8140)
15 to 17 DOI	r = 0.203746 (*P* = 0.0297)	r = 0.253231 (*P* = 0.0621)	r = 0.088693 (*P* = 0.5041)
13 to 17 DOI	r = 0.137836 (*P* = 0.1436)	r = 0.328385 (*P* = 0.0144)	r = 0.064443 (*P* = 0.6277)

1 The LTRT eggs were incubated at 37.5°C from 0 to 12 DOI, and then at 35.6°C from 12 to 18 DOI.

### Hatch

**Figure 7:**
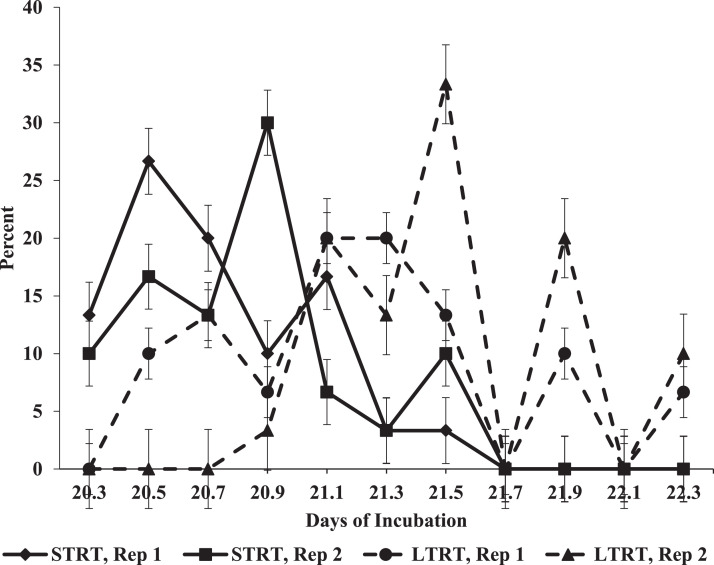
Hatch of viable chicks as a non-cumulative percentage of the number of eggs determined to contain live embryos at 12 d of incubation (DOI) in each individual replicate incubator in the 37.5°C (STRT) and 35.6°C (LTRT) treatments between 20.3 and 22.3 DOI (487 to 535 h of incubation). Eggs in the STRT and LTRT were incubated at 37.5°C from 0 to 12 DOI.

**Figure 8:**
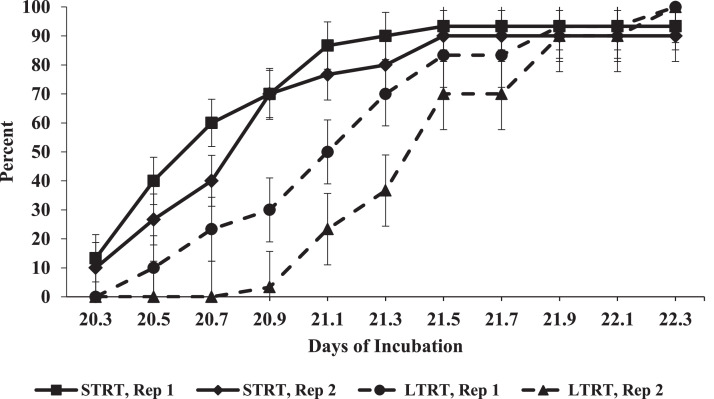
Hatch of viable chicks as a cumulative percentage of the number of eggs determined to contain live embryos at 12 d of incubation (DOI) in each individual replicate incubator in the 37.5°C (STRT) and 35.6°C (LTRT) treatments between 20.3 and 22.3 DOI (487 to 535 h of incubation). Eggs in the STRT and LTRT were incubated at 37.5°C from 0 to 12 DOI.

The noncumulative patterns of HVC in each replicate incubator in each temperature treatment are shown in Figure 7. The highest HVC values occurred between 20.5 and 21.1 DOI in the incubators belonging to the STRT, whereas the highest HVC values in the LTRT occurred between 21.1 and 21.9 DOI. Furthermore, chicks stopped hatching in the STRT by 21.7 DOI (521 h of incubation), whereas chicks in the LTRT continued to hatch up to 22.3 DOI (535 h of incubation) (Figure 7). There was a marginally significant (*P* = 0.059) main effect of treatment for HT. Mean HT in the STRT was 20.8 DOI (498 h of incubation), and in the LTRT was 21.4 DOI (513 h of incubation). These results indicate that hatchlings in the LTRT experienced an approximate 14 to 19 h delay in hatch in comparison to those in the STRT. The cumulative values of HVC in each replicate incubator in each temperature treatment are shown in Figure 8. Across the replicate incubators, cumulative HVC at 22.3 DOI in the STRT was 93.3% and was 100% in the LTRT. In the STRT, one live chick was classified as a cull and 3 eggs (one in one replicate incubator and 2 in the other) failed to hatch. Examination of the 3 residue eggs confirmed that one was an early, (0–7 DOI) one was a middle (7–14 DOI), and one was a late (14–22.3 DOI) incubation embryo mortality. Hatchling BW was significantly greater in the STRT in comparison to that in the LTRT (Table 1).

## DISCUSSION

Egg temperature is influenced by IAT (French, 1997). Prior to 18 DOI, the pattern of changes in egg temperature are relatively consistent with those of IAT (Romijn and Lokhorst, 1955). However, Romijn and Lokhorst (1956) observed that egg temperature does not rise above that of IAT before 10 DOI, and French (2009b) has stated that during the first half of incubation, the temperature inside the egg can be lower than that of the IAT. During the first days of incubation, egg temperature is lower than that of IAT because evaporative heat loss from the egg largely exceeds the amount of heat produced by the embryo (Romijn and Lokhorst, 1960). Nevertheless, in conjunction with the production of metabolic heat by the developing embryo, internal egg temperature increases above that of the IAT after the midpoint of incubation (French, 1997). This is in accordance with Romjin and Lokhorst (1956) who found that egg temperature surpasses that of IAT after 10 DOI due to a rise in the level of heat production by the embryo. Similarly, in the current study, ACT increased linearly and by 15 DOI significantly exceeded IAT.

Although EST was not monitored in the current study, ACT would be expected to be higher than EST but would follow the same pattern of change with embryonic age. These expectations are based on findings in the previous study by Peebles et al. (2012) in which a comparative evaluation of air cell and eggshell temperature measurements was made in broiler hatching eggs during incubation. In that study, it was further concluded that ACT readings provide a closer and more accurate estimation of actual ET. Mean IAT across replicate incubators between 12 and 18 DOI in the STRT was higher by approximately 1.40°C than that in the LTRT in the current study. Hamidu et al. (2018) incubated Ross 708 broiler hatching eggs between 15 and 21.5 DOI at an IAT of either 36.0, 36.5, 37.0, or 37.5°C, and found that EST increased as IAT increased, and that EST was higher at 37.0 or 37.5°C, than at 36.0 or 36.5°C. Correspondently, even though there were similar linear increases in ACT over time in both the STRT and LTRT, mean ACT in the STRT was approximately 1.37°C higher than mean ACT in the LTRT from 12 to 21 DOI in the current study. Although mean ACT was higher in the STRT than in the LTRT, it was noted that the pattern of differences between mean IAT and ACT across temperature treatment during incubation mirrored those of the STRT, but not those of the LTRT. Furthermore, IAT and ACT were positively correlated across temperature treatment and within the STRT but were not correlated in the LTRT. These mean comparisons across and within temperature treatment and the correlation results for IAT and ACT indicate that there was a close association between IAT and ACT in the STRT but not in the LTRT. It has been proposed by Romjin and Lokhorst (1955) that a protective reaction to IAT cooling through increased skeletal muscle activity and carbohydrate metabolism can occur in embryos despite their poikilothermic characteristics. This would provide an increase in the generation of metabolic heat that would counteract the effects of IAT cooling and that would subsequently increase ACT. This reaction would likewise lead to an uncoupling of the tight relationship between IAT and egg temperature and may help explain the lack of a close association between IAT and ACT in the LTRT.

As a byproduct of the metabolism of lipids, the amount of metabolic water in the egg increases as the embryo develops (Ar, 1991). The rate of water loss from an egg is dependent upon the water vapor pressure gradient that exists across the shell. The production of metabolic water in addition to a rise in egg temperature, in response to an increased output of metabolic heat by the embryo, causes the water vapor inside the egg to become even greater relative to that in the incubator, which subsequently leads to a greater rate of water loss from the egg (Ar, 1991). A higher EST during the later stages of incubation has been observed to increase the rate of chick development (Lourens et al., 2007), and Van den Brand et al. (2019) observed that the PEWL of layer hatching eggs between 14.5 and 18.5 DOI was greater when they had an EST of 38.9°C than an EST of 36.7°C. Therefore, although no other telemetric studies have been conducted to directly compare ACT to PEWL to further confirm the effects of ACT on PEWL at various IAT regimens, the significant positive correlation results between PEWL and ACT throughout the 13 to 17 DOI period in the STRT and their association with the correlation results between IAT and ACT suggests that changes in ACT in response to IAT influenced those of PEWL in the STRT but not in the LTRT. The embryos belonging to the STRT may have developed more rapidly with higher rates of metabolism and heat production leading to a higher water vapor pressure inside their eggs than embryos in the LTRT, thereby causing the PEWL of the eggs in the STRT to have been affected to a greater extent than those in the LTRT. Furthermore, a significantly greater hatchling BW in the STRT group in comparison to those in the LTRT group in the current study would support the suggestion of a higher rate of development in the embryos that were provided a 37.5 °C IAT between 12 and 21 DOI.

It is well documented in the literature that changes in IAT can affect HT (Barot, 1937; Lourens et al., 2005; Van der Pol et al. 2014; Almeida et al. 2016; Maatjens et al., 2016; Hamidu et al., 2018; Van den Brand et al., 2019). Upon incubating Cobb 500 broiler hatching eggs from 13 DOI until full hatch (21.7 DOI) at either 36.0, 37.5, or 39.0°C, Almeida et al. (2016) observed that HT was significantly longer in the 36.0°C treatment in comparison to the either the 37.5 or 39.0°C treatments. Similarly, in the current report, when compared to the STRT, the HT of the Ross 708 broiler hatching eggs was significantly delayed by 14 to 19 h in the LTRT. Conversely, the HVC results of the 2 studies differed. Almeida et al. (2016) reported that the hatchability of the fertile Cobb 500 eggs used was significantly higher in the 39.0°C treatment in comparison to either the 37.5 or 36.0°C treatments, whereas the HVC of the Ross 708 eggs in the LTRT of this study was 6.7% higher than that in the STRT. Upon comparing these studies, it is suggested that initiating a 35.6°C IAT regimen at 12 DOI might uniquely allow for better subsequent hatch results in the Ross 708 broiler hatching eggs, and that differences in the results of these 2 studies may be related to differences in the incubational growth rates and metabolic profiles of the embryos from the 2 different broiler strains. This potential explanation is further supported by the results of Nyuiadzi et al. (2017), who reported an intermediate effect when no significant differences were observed in the hatchability rates of Ross 308 broilers incubated at either 37.6 or 36.6°C from 10 to 18 DOI. Based on the above results, it is suggested that the Ross 708 embryo may receive some benefit from a lower IAT that would provide for a greater loss of metabolic heat to accommodate its rapid rate of development.

In conclusion, lowering IAT from 37.5°C (STRT) to 35.6°C (LTRT) between 12 and 21 DOI lowered ACT, increased HT, and decreased hatchling BW, indicating a subsequent effect on embryo growth and metabolism. Nevertheless, mean HVC in the LTRT was 6.7% higher than that in the STRT. Furthermore, the significant positive correlations between IAT and ACT and between ACT and PEWL that occurred exclusively in the STRT, suggested that changes in ACT in response to IAT influenced PEWL only in the STRT. Although lowering IAT at 12 DOI to 35.6°C may improve hatchability, an associated delay in hatch and a decrease in hatchling BW may not be commercially justifiable. Future research examining the effects of IAT other than 35.6 and 37.5°C on ACT and broiler embryo growth and metabolism in a forced draft incubator is warranted.
